# Adaptive Salvage Radiation Therapy for Stage IIIB Prostate Adenocarcinoma Status Post-prostatectomy

**DOI:** 10.7759/cureus.70280

**Published:** 2024-09-26

**Authors:** Mannat Bedi, Steven Miller, Jay Burmeister, Nagaraju Mogili, Ramesh Boggula

**Affiliations:** 1 Department of Oncology, Wayne State University School of Medicine, Detroit, USA

**Keywords:** psa elevation post-radical prostatectomy, serum psa level, prostate-specific antigen (psa), adaptive radiation therapy, external beam radiation

## Abstract

The prostate and post-prostatectomy surgical bed can shift in anatomical position due to changes in the bladder and rectum size. This mobility of the prostate and prostatic bed, along with that of the bladder and rectum, poses a challenge in devising a single radiation therapy plan capable of delivering the desired dose to each organ across all treatment fractions. Adaptive radiation therapy (ART) represents a significant advancement in cancer treatment. The Ethos^TM^ ART system (Varian Medical Systems, Inc., Palo Alto, CA) streamlines the adaptive therapy workflow, enabling the efficient creation of superior radiation treatment plans based on anatomical orientation at the time of treatment. This case report aims to discuss how the online ART workflow was utilized in a 72-year-old male with recurrent prostate cancer post-prostatectomy. Our results demonstrated the advantage of having the flexibility to choose between scheduled and adapted plans based on daily images, providing improved radiotherapy plan quality for prostate cancer treatment post-prostatectomy.

## Introduction

Prostate cancer is the most common cancer to afflict men in the United States and the second deadliest after lung cancer [1]. The diagnosis of prostate cancer varies, as several factors, such as age and race, influence the risk and prognosis [2]. Men over the age of 65 years of age and African American men have higher incidence rates of prostate cancer as compared to other populations [2]. Further, one in eight men will receive a prostate cancer diagnosis in their lifetime, so understanding and creating novel treatment options for both localized and metastatic prostate cancer is of paramount importance [1]. Huang et al. noted that the mobility of the prostate, bladder, and rectum makes it challenging to create a single radiation therapy plan that will provide the desired dose to each organ for all treatment fractions [3]. Additionally, Chen et al. observed that variations in bladder volume correspond to proportional alterations in the radiation dose absorbed by the bladder, suggesting that bladder volume is a crucial parameter to assess before and during radiation therapy [4].

During each adaptive radiation therapy (ART) session, the Ethos^TM^ system (Varian Medical Systems, Inc., Palo Alto, CA) produces automated artificial intelligence-based contouring of structures. Subsequently, a clinician is required to review and approve these contours, adjusting them manually as necessary. The system then generates two plans for each session: a "scheduled" and an "adapted" plan. The scheduled plan is generated by recalculating the dose distribution that would result if the initial treatment plan was delivered to the current cone beam CT (with the session contours), and the adapted plan is created by optimizing the original treatment plan to meet the original clinical dosimetric goals. These plans are typically compared, and the clinician selects the preferred treatment based on clinical goals for that session and any patient-specific factors that might affect this decision. This case report aims to discuss how online adaptive therapy workflow was used in a 72-year-old male with recurrent prostate cancer post-prostatectomy.

## Case presentation

The patient is a 72-year-old male who initially underwent a prostatic biopsy due to an elevated prostate-specific antigen (PSA) level of 8.4 ng/dL. The prostatic biopsy was consistent with a Gleason score of 3+3=6 adenocarcinoma of the prostate. He subsequently had a radical prostatectomy, and pathology was correlated with a Gleason score of 4+4=8 adenocarcinoma of the prostate with lymphovascular space invasion with a positive apical margin.

**Figure 1:**
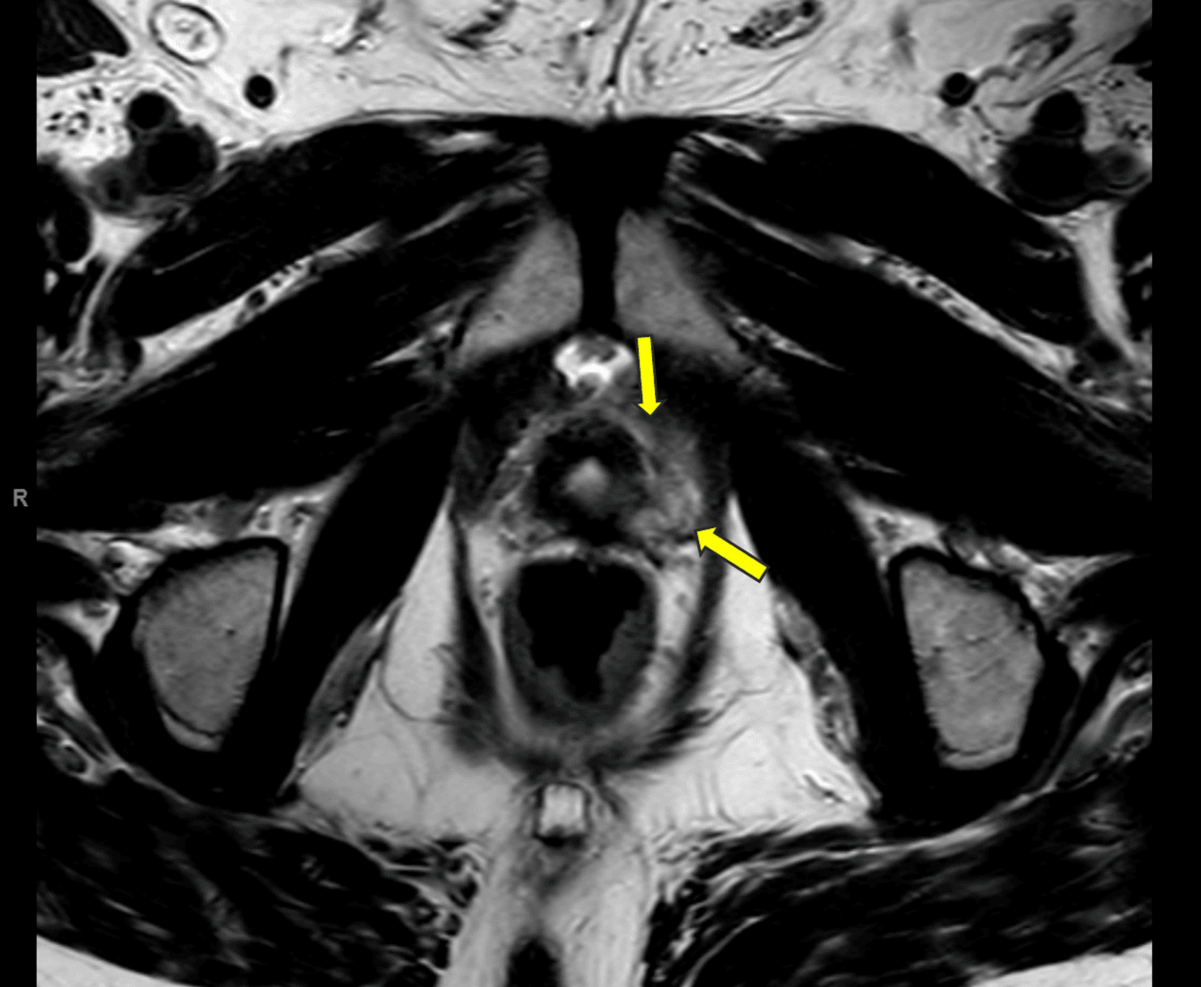
Axial T2 TSE MRI displaying loss of T2 hypointense signal in the left lateral aspect of the bladder neck The yellow arrows point to the area of recurrent disease adjacent to the bladder neck. TSE: turbo spin echo, MRI: magnetic resonance imaging

The postoperative PSA immediately after surgery was 0.05 ng/mL. He declined adjuvant radiation therapy at that time, and his PSA continued to increase. A prostate-specific membrane antigen (PSMA) PET scan was performed once his PSA reached a level of 2.47 ng/mL, which revealed no evidence of disease. A pelvic MRI revealed an area of focal arterial hyperenhancement along with a T2 hypointense signal in the left lateral aspect of the bladder neck and adjacent perivesical region with a corresponding area of hyperintense signal on the diffusion-weighted imaging as shown in Figure 1.

No enlarged pelvic lymph nodes or suspicious osseous lesions were noted.

A biopsy of the bladder neck lesion was performed, and pathology was consistent with prostatic adenocarcinoma, with a Gleason score of 7 (3+4), grade group 2, involving two cores and 30% of the total core tissue. An additional biopsy demonstrated a prostatic adenocarcinoma, Gleason score 7 (3+4), grade group 2, involving one fragment, comprising 10% of the total core tissue, and Gleason pattern 4 involved 20% of the total tumor volume.

The patient underwent a course of salvage radiation therapy using intensity-modulated radiation therapy (IMRT), with the treatment volume including the pelvic lymph nodes, prostatic bed, and bladder neck lesion, followed by a simultaneous integrated boost to the bladder neck lesion and the prostatic bed. A total of 4500 centigray (cGy) in 25 fractions was delivered to the pelvic lymph nodes and prostatic bed, as well as the bladder neck lesion identified on MRI, over a span of 35 days. The prostatic bed received an additional 11 fractions of 180 cGy each, culminating in a total dose of 6480 cGy delivered in 36 fractions. In addition, the MRI-identified bladder neck lesion received a boost of 2420 cGy in 11 fractions of 220 cGy over 19 days, using a simultaneous integrated boost technique, resulting in a total dose of 6940 cGy to the bladder neck lesion. The equivalent dose in two Gray fractions (EQD2) for prostatic tissue, using an alpha/beta ratio of 3 [5], was 6837 cGy for the bladder neck lesion. The dose to the bladder neck lesion was marginally lower than the recommended doses cited in the literature due to concerns regarding urethral toxicity [6].

**Figure 2:**
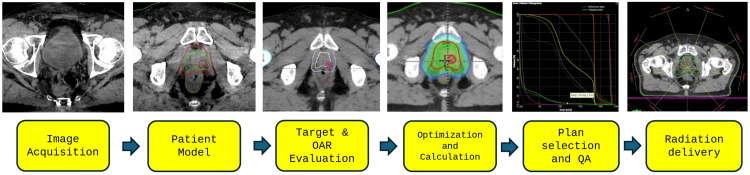
Workflow of adaptive radiotherapy The diagram shows the key steps involved in the adaptive radiotherapy process, including initial imaging, automatic generation of contours, evaluation of target and OAR, plan adaptation based on daily anatomical changes, plan selection (scheduled and adapted), pre-treatment QA, and delivery of radiation. OAR: organs at risk, QA: quality assurance

**Figure 3:**
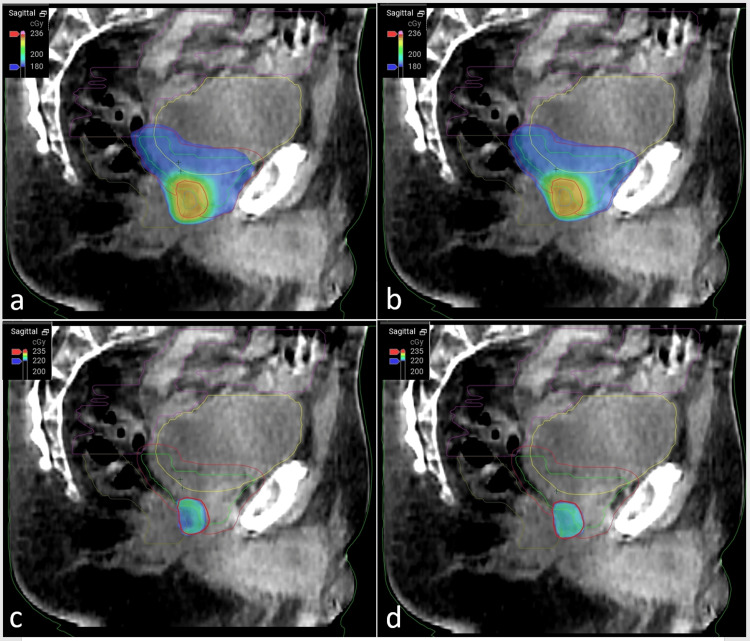
Example of a treatment fraction showing minimal daily variation in the prostatic bed and surrounding critical organs Scheduled (not adapted) plans for this treatment are displayed in panels a and c, while the associated adapted (re-optimized) plans are shown in panels b and d. The use of the scheduled plan resulted in adequate coverage of the PTV. PTV: planning target volume

**Figure 4:**
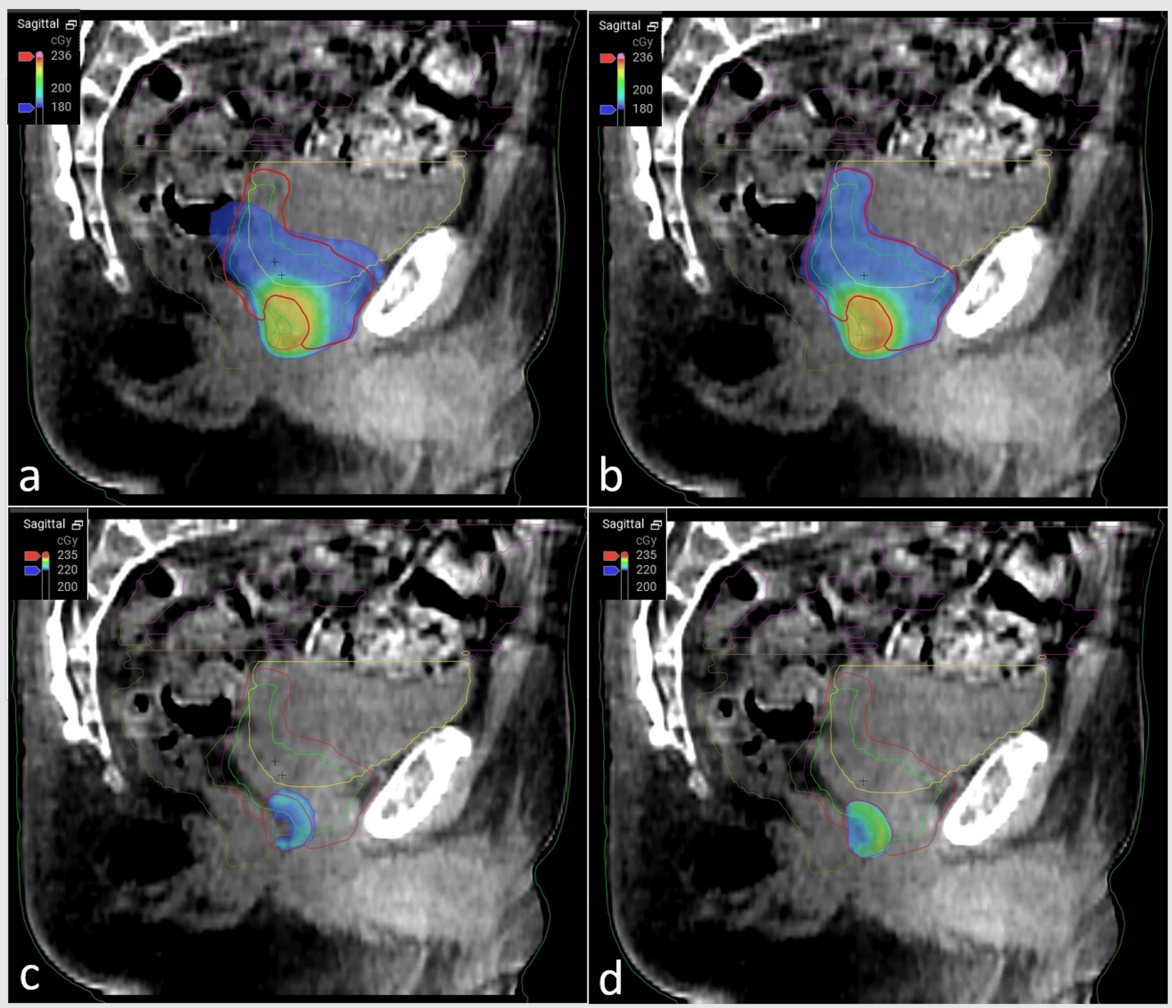
Example of a treatment fraction demonstrating the substantial daily variation in the prostatic bed and surrounding critical organs Scheduled (not adapted) plans for this treatment are displayed in panels a and c, while associated adapted (re-optimized) plans are shown in panels b and d. The use of the scheduled plan would have resulted in suboptimal PTV coverage and a higher delivered dose to the bowel. PTV: planning target volume

An online adaptive radiation plan was developed for this patient's treatment, and the treatment plan was adjusted for each fraction of the boost treatment. The typical workflow for the adaptive radiotherapy process is shown in Figure 2. The plan selection for a given day was based on meeting several clinical goals, not just the clinical goal set for target coverage. On a daily basis, plan quality was determined by the evaluation of doses received by both target and normal tissue structures while accounting for the accuracy and uncertainty of the target delineation based on the quality of the cone beam CT (CBCT) scan. The selection of an adaptive radiation therapy plan includes an extra step for the independent verification of the new plan using secondary calculation software, such as Mobius3D (Varian Medical Systems). Zhao et al. showed that Mobius3D provides a safe and reliable method for verifying Ethos-adapted plans [7]. Whenever similar clinical goals were achieved for both the plans (scheduled and adapted), the clinician preferred the scheduled plan since it does not require additional pre-treatment quality assurance and, therefore, can be completed more efficiently. This minimizes both clinical resources and time during which the patient's anatomy may change. Figure 3 represents a treatment fraction with minimal daily anatomical variation, leading to the selection of the scheduled plan for this fraction. Figure 4 displays a session with significant variation in which an adapted plan was chosen for treatment. Our treatment approach included both scheduled and adaptive sessions, reflecting real-world clinical practice where an adapted plan was not always used. This study compared various dose-volume histogram (DVH) parameters between the original, scheduled, adapted, and combined plans.

## Discussion

This study evaluated the cumulative results from various options for the delivery of an adaptive course of radiotherapy. The options were as follows: (1) delivering the scheduled treatment plans throughout the entire radiation treatment course, (2) delivering the adapted treatment plans throughout the entire radiation treatment course, or (3) delivering the actual clinician's choice treatment course, which included both scheduled and adapted plans, referred to here as the "combined" option. Our results show that the clinician's choice, a combination of scheduled and adapted treatment plans, achieved target coverage comparable to treatment with only adapted plans. The DVH parameters for the original, adapted, scheduled, and combined plans are summarized in Table 1. For example, the average volume that received 98% of the dose (V98%) for the planning target volume that received 6840 cGy (PTV6480) and the planning target volume that received 6920 cGy (PTV6920) in the combined plans was 97.7%±3.7% and 98.1%±4.6%, compared to 99.9%±0.1% and 99.7%±0.2%, respectively, in the adapted plans only. While adjusting the plan to maintain target coverage, it is not uncommon for adaptive plans to deliver a higher dose to some critical structures than an associated schedule plan. For example, a decrease in rectal volume can shift the PTV posteriorly. The scheduled plan is not able to account for this shift and would, therefore, deliver the prescription dose to the original location of the PTV. In this case, the rectum will have moved farther from the high-dose region, while the bladder will have moved closer, resulting in a lower-than-expected dose to the rectum and a higher-than-expected dose to the bladder. Conversely, the adaptive plan shifts the dose distribution to match the PTV, thus delivering a higher dose to the rectum and a lower dose to the bladder than the scheduled plan in this situation. This is evident in Table 1, where the rectal dose was 20 cGy higher in the adapted plans, while the bladder dose was 15 cGy lower compared to the scheduled plan.

**Table 1:** Comparison of clinical goals achieved across the original, scheduled, and adaptive plans, and a combination of scheduled and adaptive plans throughout the treatment The original plan is based on the simulated CT, the scheduled plan applies it daily without dose adjustments, and the adapted plan adjusts the dose in real time to account for anatomical changes. The CTV6840 included the prostatic bed, and the CTV6920 included the disease noted on the MRI. The PTV for the 6480 cGy and the 6920 cGy dose included an additional 5 mm expansion around the CTV. CT: computed tomography, CTV: clinical target volume, cGy: centigray, CTV6840: CTV receiving 6840 cGy, CTV6920: CTV receiving 6920 cGy, MRI: magnetic resonance imaging, PTV: planning target volume, V98%: volume receiving 98% of the radiation dose, V100%: volume receiving 100% of the radiation dose, OARs: organs at risk, DVH: dose-volume histogram, SD: standard deviation

Targets/OARs	DVH metric	Original plan	Scheduled course		Adapted course		Combined course	
			Average	SD	Average	SD	Average	SD
CTV6480	V98% (%)	100	98.3	2.8	100.0	0.0	99.8	0.6
CTV6920	V98% (%)	100	99.9	0.3	100.0	0.0	100.0	0.0
PTV6480	V98% (%)	99.9	93.2	6.0	99.9	0.1	97.7	3.7
	V100% (%)	99.5	90.3	7.1	99.6	0.1	96.5	4.8
PTV6920	V98% (%)	99.6	93.6	6.2	99.7	0.2	98.1	4.6
	V100% (%)	95.2	77.8	12.2	95.9	1.0	91.3	8.2
	Dmax (%)	106.5	105.1	0.7	105.6	1.6	105.5	1.1
Bladder	Dmax (cGy)	2207	2240.0	136.4	2225.0	112.8	2234.0	112.7
Bowel	Dmax (cGy)	2073	1832.0	557.4	1921.0	356.9	1932.0	277.5
Rectum	Dmax (cGy)	2504	2477.0	18.3	2497.0	19.1	2489.0	17.8

The frequent and substantial changes in the shape and orientation of targets and surrounding normal tissues involved in the treatment of prostate cancer make it an excellent case to demonstrate the benefit of online adaptive radiation therapy. Siciarz et al. compared multiple adaptive radiation therapy strategies in treating prostate cancer patients and found that daily online adaptation improved dosimetric plan quality for targets and normal tissues compared to offline ART using the patient's previous CT scans or non-adaptive approaches [8]. Meyers et al. demonstrated a statistically significant decreased PTV and significant reductions in multiple normal tissue dose metrics from the application of offline ART for post-prostatectomy prostate cancer treatment [9]. Similarly, based on the results observed in the case presented here, we expect substantial improvements in this patient cohort using an ART workflow.

## Conclusions

In summary, online adaptive radiation therapy workflow combining scheduled and adaptive sessions for post-prostatectomy prostate adenocarcinoma offers promising potential improvements in clinical efficacy, including reduced doses to OARs and improved PTV coverage.
